# Sensitivity analyses for improving sulfur management strategies in winter oilseed rape

**DOI:** 10.1371/journal.pone.0204376

**Published:** 2018-09-20

**Authors:** Emilie Poisson, Sophie Brunel-Muguet, François Kauffmann, Jacques Trouverie, Jean-Christophe Avice, Alain Mollier

**Affiliations:** 1 UMR 950 EVA (Ecophysiologie Végétale et Agronomie), Normandie Université, UNICAEN, INRA, SFR Normandie Végétal, Caen, France; 2 UMR 6139 Laboratoire de Mathématiques Nicolas Oresme, Normandie Université, UNICAEN, CNRS, Caen, France; 3 ISPA, Bordeaux Sciences Agro, INRA, Villenave d’Ornon, France; Universidade de Lisboa Instituto Superior de Agronomia, PORTUGAL

## Abstract

Because sulfur (S) depletion in soil results in seed yield losses and grain quality degradation, especially in high S-demanding crops such as oilseed rape (*Brassica napus* L.), monitoring S fertilisation has become a central issue. Crop models can be efficient tools to conduct virtual experiments under different fertilisation management strategies. Using the process-based model SuMoToRI, we aimed to analyse the impact of different S fertilisation strategies coupled with the variablility observed in major plant characteristics in oilseed rape i.e. radiation use efficiency (RUE), carbon (C) allocation to the leaves (β) and specific leaf area (SLA) on plant performance-driven variables encompassing total biomass (TDW), S in the photosynthetic leaves (QS_mobile.GL_) and leaf area index (LAI_GL_). The contrasting S supply conditions differed in the amount of S (5 levels), and the timing of application (at bolting and/or at flowering, which included a fractioned condition). For this purpose, we performed a global sensitivity analysis (GSA) and calculated two sensitivity indices i.e. the Partial Raw Correlation Coefficient (PRCC) and the Sobol index. The results showed that whatever the timing of S supply, TDW, LAI_GL_ and QS_mobile.GL_ increased as S input increased. For a given S supply, there was no difference in TDW, LAI_GL_ and QS_mobile.GL_ between a single and a fractioned supply. Moreover, delaying the supply until flowering reduced the TDW and LAI_GL_ whereas QS_mobile.GL_ increased. Results showed that RUE had the greatest impact on TDW under all levels of S supply and all application timings, followed by β and SLA. RUE mostly impacted on QS_mobile.GL_, depending on S supply conditions, whereas it was the parameter with the least impact on LAI_GL_. Ultimately, our results provide strong evidence of optimised S fertilisation timings and plant characteristics that will guide producers in their agricultural practices by using specific varieties under constrained S fertilisation strategies.

## Introduction

Crop models can be efficient tools to simulate and analyse agricultural practices, such as the impact of fertiliser management strategies on crop performances [1–6]. They provide a framework to untangle interactions between plants and their environment, and often deliver quantitative outputs indicating thresholds to target specific objectives. In the current context of sustainable agriculture, which implies reduction of fertiliser inputs, modelling experiments are less expensive and easier to set up than field or greenhouse experiments but allow fertilisation constraints to be tested. In addition, most crop models have been used extensively to analyse the impacts of on-going global changes and to project crop yields worldwide [7–11].

Modelling approaches to assist crop management in low input systems can target fertilisation strategies (amount, timing and fractioning) or plant characteristics, which in both cases aim to alleviate the negative impact of decreased resources. The advantage of process-based models is that (i) they clarify plant characteristics and central processes such as photosynthesis, nutrient uptake, assimilation or remobilisation by means of equations and parameters, and (ii) they take into account the variability of plant features. Therefore, exploring the effects of the variation in plant parameters on model outputs can be achieved with sensitivity analyses (SAs) [12]. SAs are usually carried out to assess the sensitivity of model outputs with respect to the inputs i.e. parameters and/or variables (due to their estimation uncertainty or inherent variability). SAs with process-based models have several objectives: (i) to explored model functioning; (ii) to improve model calibration by adding or deleting processes (i.e. parameters and equations) which define the level of complexity in the model according to their impacts on output variations [13]; (iii) to quantitatively estimate the extent of the impacts of input variables (representing resource allocation for instance) on crop performance; and (iv) to identify the most influential plant parameters (representing plant features) according to their effect on output variations, and thus to help defining ideotypes (or virtual genotypes). Two categories of SAs are usually carried out. First, the local approach (LA) aims to study the variability of outputs induced by the small variation of a single parameter and/or variable (input) to the point when an estimate is chosen while keeping the other parameters fixed [14]. Second, a global sensitivity analysis (GSA) considers in contrast the whole variation range or domains of the inputs, which allows outputs to be analysed with regard to the combined variability [14]. GSA are commonly used with complex models where the conventional method (LA) would fail to represent the interactions between the numerous parameters and input variables [15].

In this study, we aimed to analyse the impact of different sulfur (S) fertilisation strategies coupled with the variablility observed in major plant characteristics in oilseed rape. Like most of the Brassica species, oilseed rape is a high-S demanding crop with recommended inputs (RI) (Terres Inovia, http://www.terresinovia.fr/colza/cultiver-ducolza/fertilization/soufre/) of about 30 kg S.ha^-1^ (corresponding to 75 kg SO_3_.ha^-1^) [16]. Sulfur is present in a wide range of metabolites such as methionine (essential amino acids (AA)) and cysteine (non-essential AA), proteins, glutathione and glucosinolates, which makes it an essential element for growth, development and resistance to abiotic and biotic stresses. The importance of tightly monitoring S fertilisation, especially in Brassica species, has emerged over recent decades when drastic environmental policies aimed at lowering industrial sulfur dioxide (SO_2_) emissions [17] and *in fine* led to an increased risk of soil S oligotrophy. Several studies in oilseed rape have indicated that low S availability can drastically impact seed yield and corresponding components (e.g. thousand seed weight, pod number, seeds per pod), seed nutritional characteristics (e.g. oil and protein contents, and fatty acid (FA) and seed storage protein (SSP) compositions) as well as physiological quality of seeds (e.g. germination rates, storage capacity) [18–20]. In this context, modelling S requirements and allocation within the plant could be a helpful approach to correct deficiencies occurring during vegetative growth and the transition between vegetative and reproductive stages which are crucial phases for S storage and thus for providing S to growing reproductive sinks [21–23]. The process-based model SuMoToRI (Sulphur Model Towards Rapeseed Improvement) [2] simulates the dynamics of crop growth and S status until the onset of pod formation. Its original feature is based on the prediction of an S-mineral pool that is assumed to remobilise to provide S requirements for growing sinks throughout the reproductive stage. The model is run with a limited number of plant parameters that refer to the potential leaf growth and carbon (C) demand by the leaves driven by climatic variables (temperature and radiation), genetic characteristics, C assimilation and S uptake as well as S allocation (among compartments) and partitioning (S-organic vs. S-mineral compounds).

The objective of this modelling analysis is to highlight the most suitable combinations of plant model parameters under contrasting fertilisation strategies which allowed high plant performances (biomass, leaf area or S content in leaves). For this purpose we performed a global sensitivity analysis (GSA) and determined two sensitivity indices i.e. the Partial Raw Correlation Coefficient (PRCC) and the Sobol index. Both indices are based on measures of importance (quantitative sensitivity indices) in contrast with coarse screening methods e.g. the Morris method [24], which is usually used to explore the behaviour of model outputs by varying a broad number of parameters, thus helping to detect the least influential ones. Because we aimed to focus on specific plant parameters, we used methods based on linear regression (i.e. PRCC) and on the decomposition of functional variance using Sobol indices. This allowed (i) estimation of the strength of linear associations between outputs and each input parameter after removing the linear effect of the other parameter (PRCC) and (ii) distinguishing and quantifying the principal and interaction effects of the parameter on the output variation using Sobol indices [25].

In our study, the GSA was performed under contrasting S supply conditions that differed in S availability (5 levels), date (at bolting or at flowering) and fractioning (once or twice during growth from the end of winter) of S inputs. Three plant parameters of the model SuMoToRI were targeted because they were shown to display variability in response to S [2] and/or different conditions that have been reported in the literature [26]. These parameters were the Radiation Use Efficiency (RUE, g DW.MJ^-1^), the Specific Leaf Area (SLA, m^2^.g DW^-1^) and the C-leaf allocation coefficient (β, dimensionless). The outcomes for the GSAs are twofold (i) ranking the targeted plant parameters according to their impacts on plant performances, that are the total biomass production, the S in the leaves available for remobilisation towards growing pods (used as a proxy of seed quality) and the leaf area index (the central variable in the model) under different S supply strategies and (ii) identifying the most suitable combinations of plant parameter values (namely ideotypes) under these contrasting S supply strategies. This will eventually help producers to adjust agricultural practices by using specific varieties under constrained S fertilisation strategies.

## Materials and methods

### Overview of the SuMoToRI model

SuMoToRI was described extensively in Brunel-Muguet *et al*. [2]. Briefly, this process-based model predicts with daily time increments, the dynamics of the leaf area index (LAI_GL_ which is the leaf area, LA_GL_, multiplied by plant density), the biomass, the S amounts and the fractions of organic and mineral S for three main plant compartments considered, namely the photosynthetic leaves, which are simplified as a single Big Leaf (BL), the fallen leaves (FL) and the rest of the plant. The model considers the three environmental factors of temperature, Photosynthetically Active Radiation (PAR, MJ m^-2^) and the amount of S taken up by the plant. The simulation period covers the end of vernalisation until the onset of pod formation. The mineral fraction in the leaves is estimated by the sulfate amount and this is used as an indicator of the potential for S remobilisation towards growing pods. The model is run with 23 plant parameters, most with generic values, which describe potential leaf expansion, C-assimilation, allocation of C and S among the three compartments and S-partitioning (mineral vs. organic).

### Simulations and sensitivity analysis procedures

The model was used to predict plant growth and S status under several S supply conditions that were expected to highlight contrasting plant behaviour simulations. Then the GSA was performed (i) to rank three plant parameters according to their respective impact on plant performance and (ii) to determine the most suitable plant parameter value combinations under these special conditions. Our underlying questions were the following: (i) what are the most impacted outputs resulting from the variations in the targeted parameters? (ii) To what extent do S fertilisation conditions modulate the impact of the parameter variations on outputs? The GA procedure consists of the following steps:

#### Step 1. Choice of targeted plant parameters and setting of their variation range

The GSA was performed on three of the 23 parameters in order to assess their impact on biomass and S-content: the Radiation Use Efficiency (RUE), the Specific Leaf Area (SLA), and the β parameter, which indicates the allocation of C-assimilates to the leaves. These parameters were chosen (i) because value variations were observed in response to S availability for RUE (variability observed in the model calibration and evaluation datasets) and for the Specific Leaf Area (SLA), and the β coefficient (with variability for the validation dataset only) (unpublished data and [2]) and (ii) according to the literature and prior experiments including those reported from other species [26–28]. Following these observations, the mean, the standard deviation, the distribution profile and the truncation thresholds (minimum and maximum) were determined for each parameter (Table 1). All three parameter distributions were assumed to be uniform (not negative). All other model parameters were assumed constant (Table 1).

**Table 1:** Model parameters and initial values.

Symbol	Definition	Value	Unit	Source
sd	Sowing density	40	Plant.m^2^	
**PAR interception**				
k	PAR extinction coefficient	0.75	m^2^ m^-2^	Bonhomme *et al*. 1982
**Potential leaf growth**				
LA_0_	Initial leaf area of photosynthetic leaves	0.015	m^2^ plant^-1^	Estimated
LA_max_	Leaf area expansion parameters	0.2	m^2^ plant^-1^	
K		872.96	°Cd^-1^	
N		6.31	dimensionless	
**C acquisition and plant offer**				
PARabs_ini_	Initial absorbed PAR	0	MJ m^-2^	Estimated
TDW_ini_	Initial total dry weight	0.576	g DW plant^-1^	
DW_FL.ini_	Initial dry weight of fallen leaves	0	g DW plant^-1^	
**RUE**	**Radiation use efficiency**	**1.6–4.6**	**g DW MJ**^**-1**^	
aLDW_FL_	Parameters of the function describing the time progression of LDW_FL_	0.0092	g DW plant^-1^ °Cd^-1^	
bLDW_FL_		0.0043	dimensionless	
**C allocation to leaves**				
**β**	**Coefficient of DW allocation to the leaves**	**0.10–0.72**	**dimensionless**	Estimated
**C demand of green leaves**				
LDW_GL.ini_	Initial dry weight of green leaves	0.448	g DW plant^-1^	Estimated
**SLA**	**Specific leaf area**	**0.008–0.034**	**m^2^ g DW**^**-1**^	
**Growth S demand**				
αGL	Parameters to estimate critical S content in GL as a function of LDWGL	5.11	mg S plant^-1^	Estimated
βGL		-0.52	dimensionless	
αrest	Parameters to estimate critical S content in the rest of the plant as a function of DWrest	1.83	mg S plant^-1^	
βrest		-0.004	dimensionless	
**Potential mobile S allocation**				
εpot	Coefficient of potential repartition of mobile S to the leaves	0.8	dimensionless	Estimated
**S uptake**				
QS_TOT.ini_	Initial total S uptake	6.78	mg S plant^-1^	Estimated
QS_GL.ini_	Initial S in green leaves	5.76	mg S plant^-1^	
QSrest_ini_	Initial S in fallen leaves	1.017	mg S plant^-1^	

Parameters in bold are used for the GSAs (RUE, β and SLA). Values range between minimum and maximum.

#### Step 2. Calculation of S uptake functions

**Fig 1:**
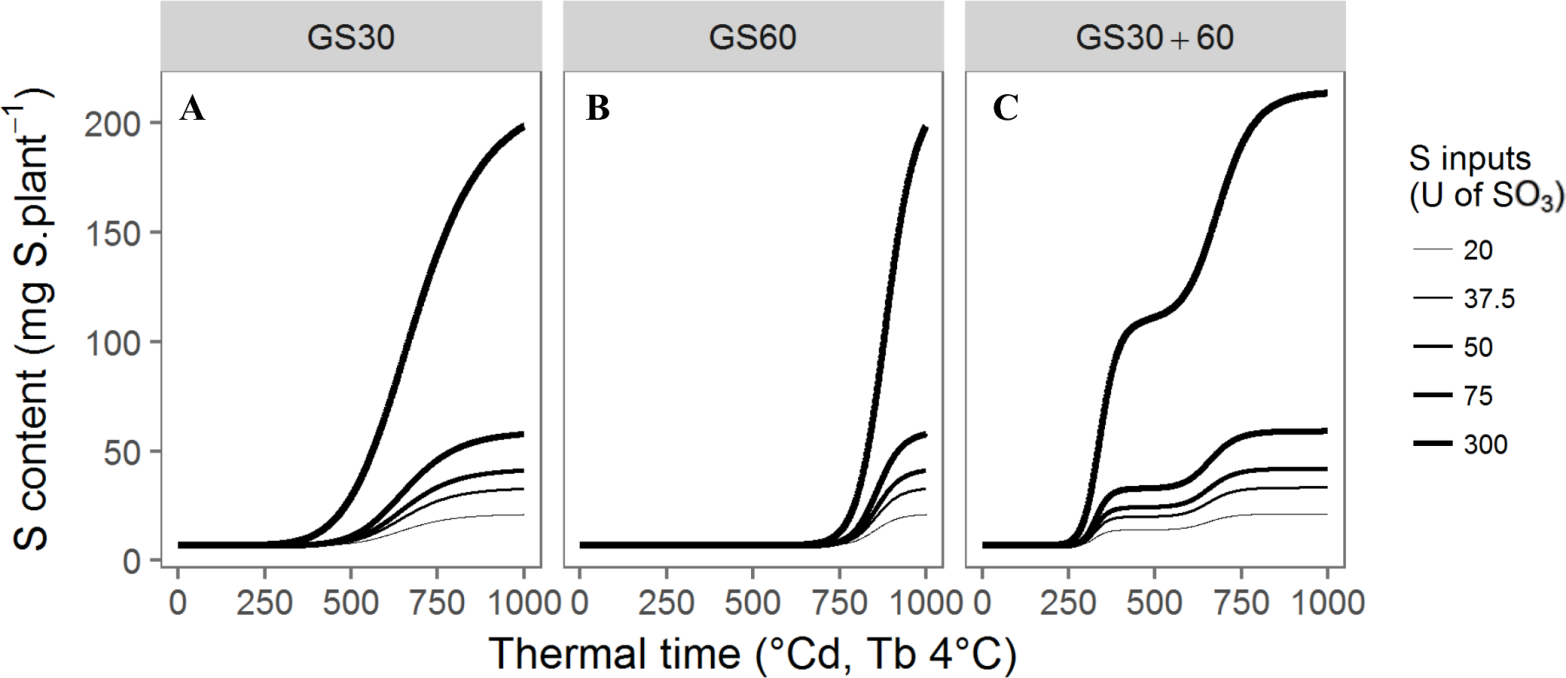
Hills’ kinetics of S uptake according to the 15 S-supply conditions. Fitted Hill’s model of S amounts as a function of thermal time (°Cd) for (A) the single S supplies at GS30, (B) at GS60 and (C) the fractioned S supplies at GS30 and GS60. Tb: base temperature.

The model was initially calibrated and evaluated with the cultivar Yudal, under two contrasting S supply conditions (High S, HS and Low S, LS) [29]. They corresponded respectively to 300 and 20 units (U) of sulfur trioxide (SO_3:_ kg.ha^-1^), which were provided throughout the crop cycle following the relative addition rate nutrient-dosing system [30,31]. Consequently, measured plant S uptake dynamics for both extreme conditions were fitted to the Hill’s model (Eq 1):

$$QS=\frac{{QS}_{max}\times {TT}^{n}}{K_{a}+{TT}^{n}}+{QS}_{ini}$$ (Eq 1)

where QS_max_ (mg S.plant^-1^), K_a_ (°Cd) and n parameters were the three plant parameters describing the uptake process, QS_ini_ (mg S.plant^-1^) the initial amount taken up by the plant at the end of vernalisation and TT, the thermal time (in °Cd) [32]. Three other intermediate S fertilisation levels were selected as follows (i) the recommended inputs (RI) for the whole crop cycle, matching 75U SO_3_, (ii) 50U SO_3_ (2/3 of the RI) and (iii) 37.5U SO_3_ (half of the RI). These amounts were supplied once at the end of winter (GS30, bolting) and 20 days later (200 °Cd, GS60, flowering) [33] (Fig 1A), or split at two times in equal amounts at GS30 and GS60 (Fig 1B) according to the following Hill’s model (Eq 2):

$$QS=\frac{{QS}_{max1}{TT}^{n1}}{K_{a1}+{TT}^{n1}}+\frac{{QS}_{max2}{TT}^{n2}}{K_{a2}+{TT}^{n2}}+{QS}_{ini}$$ (Eq 2)

The Hills’ model parameters were determined for the 15 S-supply conditions i.e. 5 amounts x 3 timings (once at GS30, once at GS60 and twice at GS30 and GS60 for the fractioned condition) (Table 2). Initial values were the same for the 15 conditions, assuming that the plants were previously grown under similar environmental conditions and optimal S nutritional supply (75 U SO_3_). Therefore, the initial amount of total S (QS_ini_) was determined relative to both HS and LS conditions by averaging the values obtained with the respective Hill’s adjustments for HS and LS. Then linear regressions were used to calculate the initial total dry weight (TDW_ini_), the initial leaf dry weight of green leaves (LDW_GLini_) and the initial amount of S in green leaves (QS_GLini_) (Table 1).

**Table 2:** Hill’s model parameters for the 15 S-supply conditions.

Total S rate supply (Units: kg.ha^-1^)	Definition	Q_Smax1_	n_1_	K_a1_	Q_Smax2_	n_2_	K_a2_
**Supply at the end of winter**							
20	Corresponding to a supply of S at the end of winter (GS30: beginning of simulation).	14.5	9.1	654	/	/	/
37.5		26.6	9.0	656	/	/	/
50		35.2	8.9	658	/	/	/
75		52.4	8.6	661	/	/	/
300		207.5	6.7	686	/	/	/
**Supply 20 days delayed**							
20	Corresponding to a supply of S 20 days after the end of winter (GS60).	14.5	24.6	854	/	/	/
37.5		26.6	24.4	856	/	/	/
50		35.2	24.2	858	/	/	/
75		52.4	24.0	861	/	/	/
300		207.5	20.8	886	/	/	/
**Fractioned S application**							
20	Corresponding to a fractioned supply of S at the end of winter and 20 days after the end of winter (GS30+GS60).	7.3	18.3	327	7.3	18.3	654
37.5		13.3	18.0	328	13.3	18.0	656
50		17.6	17.7	329	17.6	17.7	658
75		26.2	17.3	330	26.2	17.3	660
300		103.7	13.3	342	103.7	13.3	685

Equations for S-uptake adjustments are given in the material and methods section.

#### Step 3. Simulations of plant performances under contrasting S-supply conditions

Simulations were performed under 15 different S-supply conditions that differed in terms of S-amount and timing of application (including one fractioned condition) (Table 2). A single set of parameter values was used except for the three targeted parameters (RUE, SLA and β), which were randomly selected within their respective distribution (Tables 1 and 2). A single climatic dataset for simulations and the GSAs was used, based on daily average temperature and radiation from 2005 to 2015 in Saint-Martin-de-Hinx (43°34′57″N, 1°16′10″W) (data from the CLIMATIK platform, https://intranet.inra.fr/climatik_v2, S1 Fig, supporting information). The dates of initialisation for the simulations were determined with two criteria i.e. temperature (10 consecutive days above 10°C) and day length (threshold of 11.35 hours), so as to closely match the conditions at the end of vernalisation to start running the model [29].

#### Step 4. Computation of the sensitivity indices: partial rank correlation coefficient

The partial rank correlation coefficient (PRCC) measures the strength of the linear associations between the output and each input parameter, after removing the linear effect of the other parameters. These rank-based measures are part of the so-called sampling-based global sensitivity analysis method. The PRCC varies between -1 and +1 and it quantifies the links between input factors and output variables as well as the direction of the relationship. We used a “Latin Hypercube Sample” (LHS) for generating the sample of parameter combinations [34], which allows the precision of the sensitivity indices to be increased. Overall 3,000 simulations (200 repetitions x 15 conditions) for each output were performed, thus allowing 200 combinations of parameters to be tested under the 15 S-supply conditions.

#### Step 5. Computation of the sensitivity indices: functional decomposition of variance with Sobol indices

The Sobol method is a variance-based method that uses a variance ratio to estimate the importance of parameters [35,36]. Two main sensitivity indices are defined for each parameter. The first order or main order of sensitivity index measures the average effect of one parameter on one model output, without taking into account the interaction effects with the other parameters. The second order expresses the sensitivity of the model to the interactions between the parameters. The sum of both indices provides the total effect index. Prior to their estimation, we used the Monte Carlo-based sampling method, which implied n (d+2) model evaluations (N) in which n = 2000 is the size of an initial Monte Carlo sample (number of repetitions) and d = 3 is the number of targeted parameters [13]. Therefore, the index estimations required 10^4^ model evaluations (N) per S supply condition and for each output. Overall, for each output variable, 10^4^ x 15 simulations were performed under the 15 S-supply conditions.

### Software

The model was run with R (version 3.4.1) [37] with additional R packages including *pse* (Latin Hypercubes), *sensitivity* (sensitivity analysis), *ggplot2*, *ggthemes* and *reshape2* (visualisation).

## Results

Three representative outputs were selected to illustrate the variations in the model inputs and these comprised the biomass (TDW), the leaf area index (LAI_GL_) and the S-remobilisation process (QS_mobile.GL_).

### Plant performances and range of variations at the onset of pod formation

**Fig 2:**
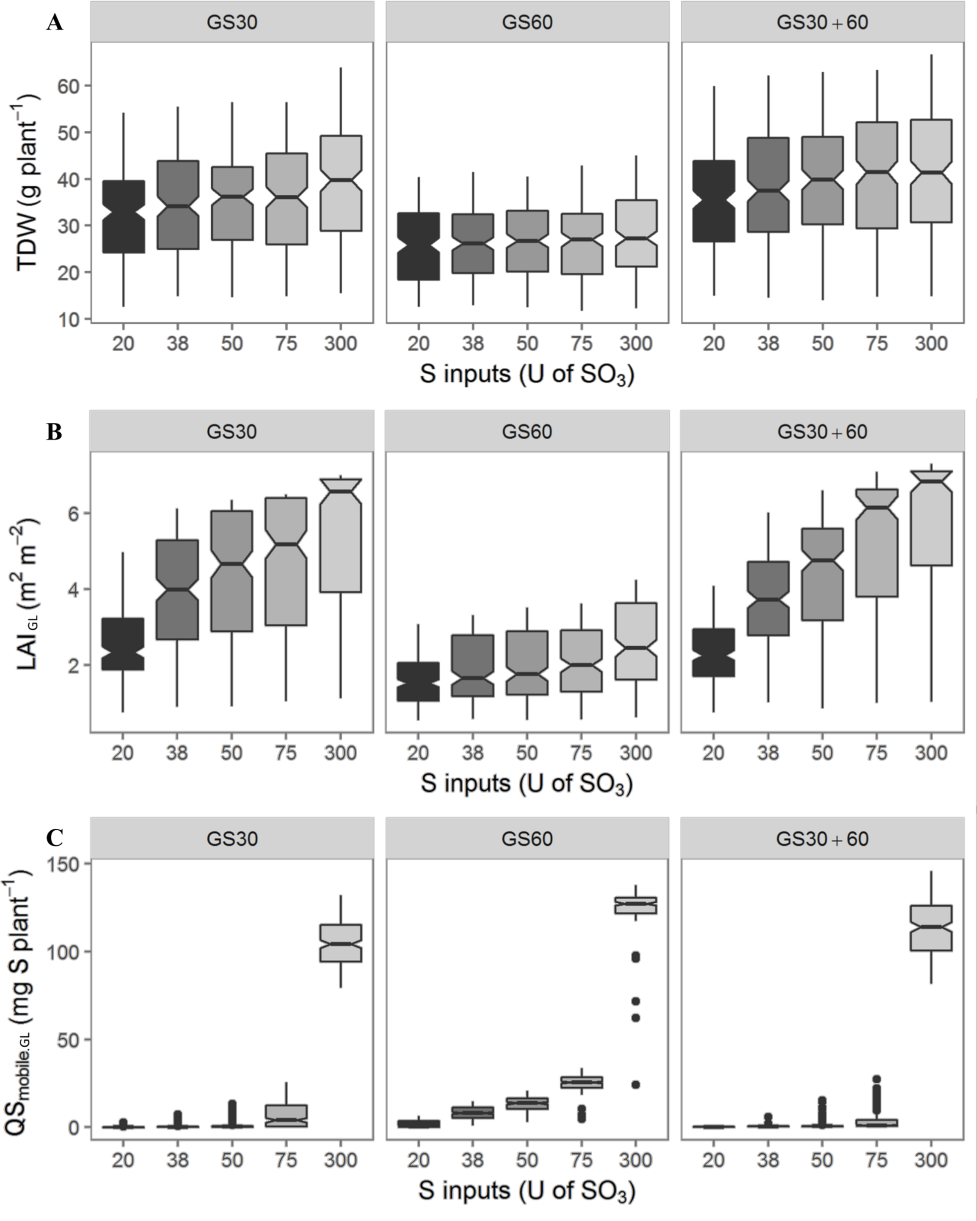
Plant performances and range of variations at the onset of pod formation for the 15 S-supply conditions according to random draws of 200 combinations of the 3 parameters tested. Variation of the TDW (g.plant^-1^) (A), LAI._GL_ (m^2^.m^-2^) (B) and QS_mobile.GL_ (mg S.plant^-1^) (C) for the 15 S-amounts x timing conditions, obtained from simulations with 200 combinations of the 3 parameters tested (RUE, β and SLA). Data are represented by a box plot and through their quartiles; the bottom and top of the box are the first or lower (Q1) and third or upper quartiles (Q3), the band inside the box is the second quartile (the median, Q2) and maximum and minimum values are at the end of the vertical bars. Extreme data are represented by point.

Fig 2 represents the extent of variation in the 3 outputs (i.e. TDW, LAI_GL_ and QS_mobile.GL_) at the onset of pod formation for the 15 S supply conditions according to random draws of 200 combinations of the 3 parameters tested (i.e. RUE, SLA and β).

Whatever the timing of the S supply (GS30, GS60 and GS30+60, which is the fractioned condition), the three outputs (TDW, LAI_GL_ and QS_mobile.GL_) increased with a higher S supply but for TDW the increase was lower (Fig 2). The median TDW ranged from 32.9–39.7 g.plant^-1^ with extreme values observed for 20U and 300U, respectively, when provided at GS30 (bolting). The S supply rate effect was stronger on LAI_GL_ when provided once at GS30 and twice at GS30+60 (with values being more than twice as high with 300U compared to 20U) and on QS_mobile.GL_, irrespective of the timing of the S supply (with the expected increase being up to 10^4^ times higher with 300U than with 20U).

For a given S supply rate there was a slight increase in the TDW, LAI_GL_ and QS_mobile.GL_ (Fig 2A, 2B and 2C) when supplying S twice at GS30+60 compared to once at GS30. However, delaying S fertilisation until GS60 noticeably reduced the TDW and LAI_GL_ (Fig 2A and 2B), regardless of the S amount, and contrasted with the increase in QS_mobile.GL_ (Fig 2C). The effect of the S amount when provided at GS60 (flowering) had less of an impact on TDW (25.8 to 27.3 g.plant^-1^) and LAI_GL_ (1.5 to 2.5 m^2^.m^-^2^^) than supplying S at GS30 (32.9 to 39.7 for TDW and 2.3 to 6.6 for LAI_GL_) or at GS30+60 (35.6 to 41.4 for TDW and 2.3 to 6.8 for LAI_GL_).

The simultaneous variation in the 3 parameters generated a high range of variation in the three outputs (Fig 2A, 2B and 2C). For TDW and LAI_GL_ the extent of variation resulting from combinations of the three parameters was more pronounced with the GS30 (bolting) and GS30+60 timings than in the GS60 timing (flowering) under all S supply rates, except in the case of LAI_GL_ where lower variation was observed with lower S amounts. For instance, the extent of variation between the minimum (min) and the maximum (max) TDW values were *ca*. 41.7 and 27.8 respectively for 20U at GS30 or GS60 (Fig 2A). For LAI_GL_, the extent of variation between max and min values was about 4.2 and 5.9 respectively for 20U and 300U at GS30 and about 2.5 and 3.6 respectively for 20U and 300U at GS60 (Fig 2B). In contrast, the extent of variation for QS_mobile.GL_ was much lower than for the other two parameters, but it was intensified under GS60 timing conditions with a similar trend for each of the five S amounts, whereas under the GS30 and GS30+60 timing conditions, the extent of variation for QS_mobile.GL_ only increased with 75U and 300U (Fig 2C).

### Impact of variation in plant parameters on plant performance and S status

To investigate the specific impact of the 3 parameters (RUE, SLA and β), global sensitivity analyses were performed. They allowed sensitivity indices (PRCC and Sobol indices) to be calculated for the three targeted outputs under the 15 conditions. Furthermore, the Sobol indices indicated and quantified the interactions between the three parameters. By doing so, we sought to rank the plant parameters according to the impact of their variations on plant performance and to see whether the S amounts x timing designs could interfere in this ranking. The results are supported by outputs that illustrate the main processes included in the model.

#### The variation in RUE was the main driver of TDW and displayed high stability across the conditions

**Fig 3:**
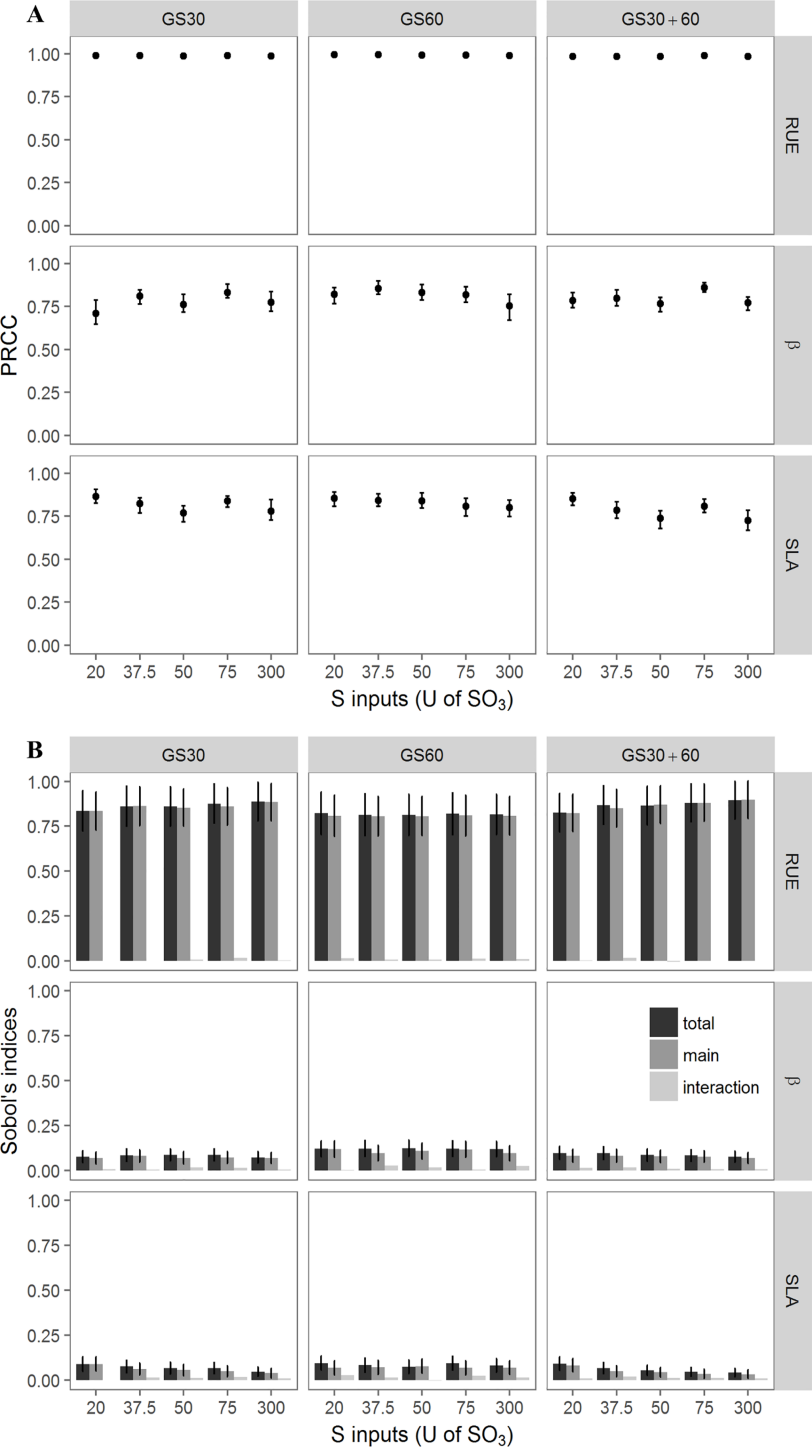
Impact of variation in plant parameters on TDW described by PRCC and Sobol indices. Impact of RUE, β and SLA calculated by PRCC (A) and by Sobol indices (B) on TDW for the 15 S-amounts x timing conditions. For Sobol indices, total indice ±SE, main indice ±SE and interaction (= total-main) are presented for each parameter and each condition (n = 15).

Plant biomass at the onset of pod formation was strongly positively impacted by the variation in RUE when PRCC values were close to 1 according to the S amounts and timing conditions (Fig 3A). Furthermore, the increase in β and SLA was positively correlated with the increase in TDW when PRCC values ranged from 0.71 to 0.86 and from 0.72 to 0.86 for β and SLA respectively under all conditions. The small range of variations in the PRCC values of RUE, β and SLA indicated that S-amounts and fertilisation timing conditions did not influence the respective impact of these three parameters on TDW. Similar conclusions could be drawn with the Sobol indices calculated for the three parameters (Fig 3B). Among the parameters, RUE had the most impact with a mean main index of 0.85 across all conditions together, which was in contrast to the much lower main index values for β and SLA. The interaction index values were low for the three parameters indicating no interaction was detected between them (Fig 3B).

#### LAI_GL_ was mainly impacted by the C-allocation to the leaves

**Fig 4:**
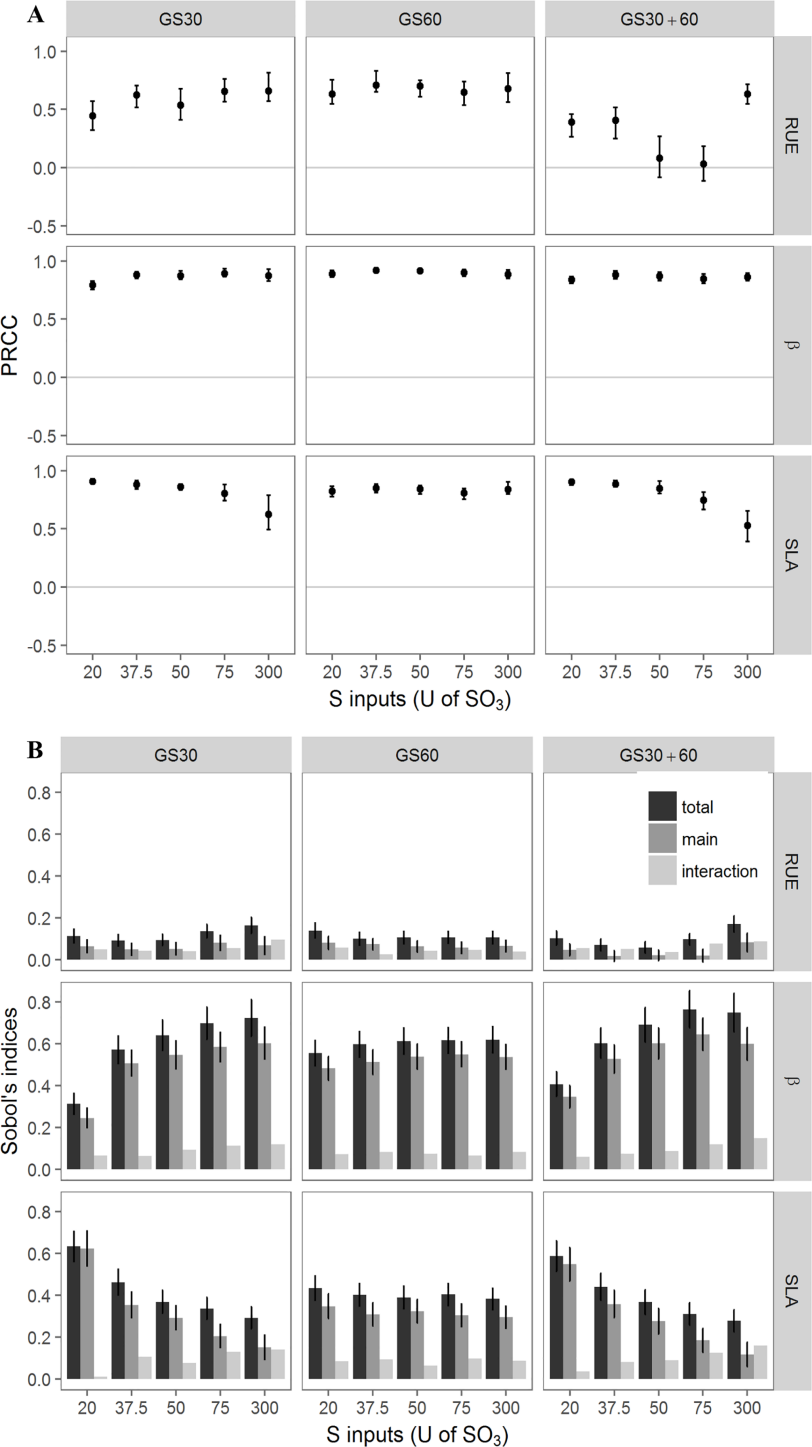
Impact of variation in plant parameters on LAI_GL_ described by PRCC and Sobol indices. Impact of RUE, β and SLA calculated by PRCC (A) and by Sobol indices (B) on LAI_GL_ for the 15 S-amounts x timing conditions. For Sobol indices, total indice ±SE, main indice ±SE and interaction (= total-main) are presented for each parameter and each condition (n = 15).

Both sensitivity indices indicated that mainly SLA and β highly influenced LAI_GL_ with different patterns depending on the levels of S fertilisation and timing conditions (Fig 4A and 4B). Regarding the PRCC indices, the mean index value of RUE reached 0.52 across all conditions together and decreased in the fractioned condition (GS30+60) with S inputs below 300U. The positive β index was the highest and was unaffected by the S amount or timing conditions. Finally, SLA index values were variable under changing S amounts in the GS30 and GS30+60 conditions, with a decrease in the index under higher S inputs. The Sobol indices confirmed that the influence of RUE variation on LAI_GL_ was the lowest, irrespective of the S amount and the timing conditions (Fig 4B). In contrast, the variation in β and SLA led to contrasting responses to the S amount and timing conditions. Under GS30 and GS30+60 conditions, the higher the S amount, the higher the impact of the variation in β on LAI_GL_. For instance, the Sobol total index under the GS30 conditions increased from 0.3 to 0.7 as the amount of S increased. In contrast, under GS30 and GS30+60 conditions, the higher the S amount, the lower the impact of SLA on LAI_GL_ whose Sobol total index decreased from 0.6 to 0.3 under the GS30 conditions. Under GS60 conditions, no effect of increased S amounts was observed on the sensitivity of LAI_GL_ to the variation in β and SLA. Interaction indices were low for the three parameters meaning that there were no tight interactions between them, regardless of the S amounts and timing conditions (Fig 4B).

#### QS_mobile.GL_ was strongly impacted by RUE and the impact of β and SLA were depending on S supply

**Fig 5:**
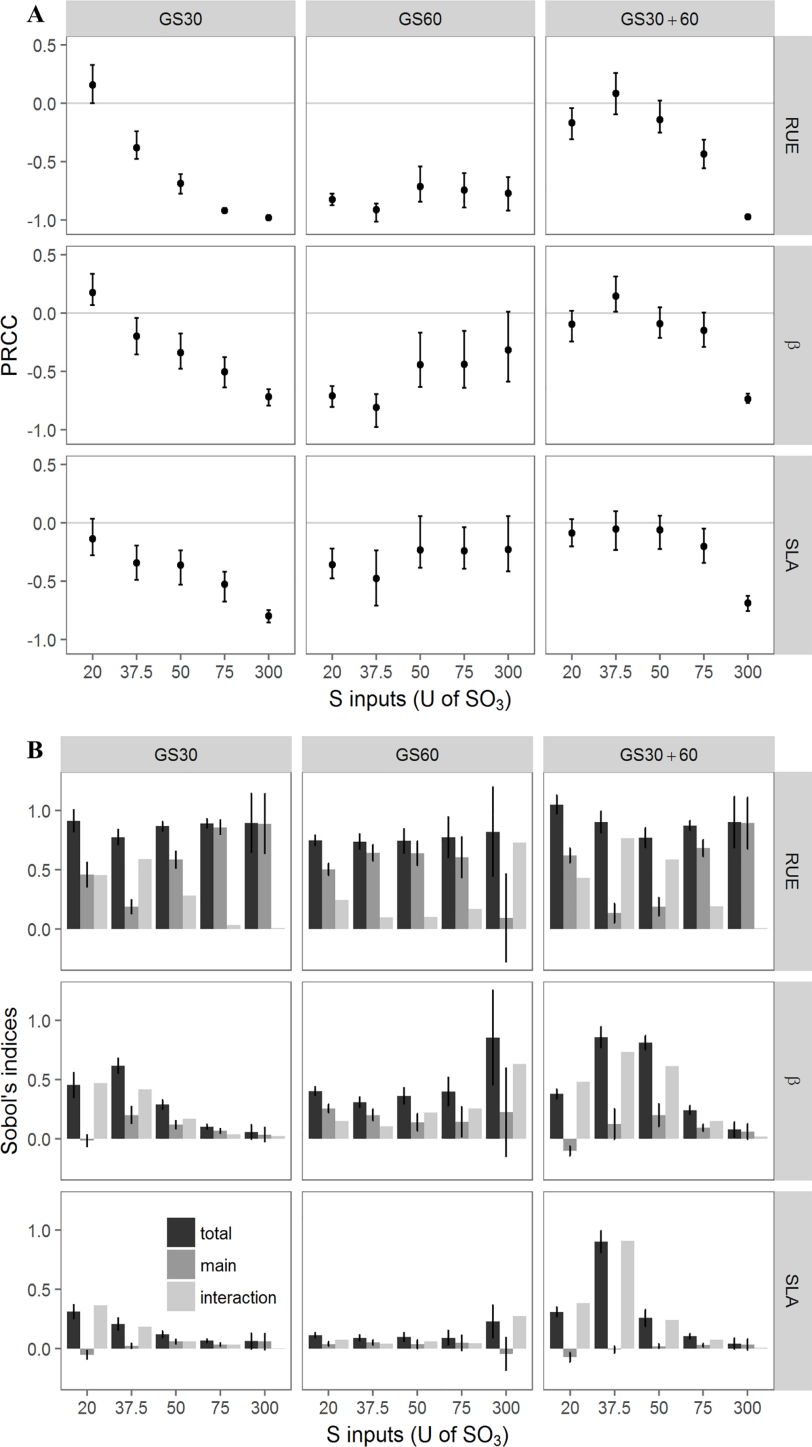
Impact of variation in plant parameters on QS_mobile_ described by PRCC and Sobol indices. Impact of RUE, β and SLA calculated by PRCC (A) and by Sobol indices (B) on QS_mobile.GL_ for the 15 S-amounts x timing conditions. For Sobol indices, total indice ±SE, main indice ±SE and interaction (= total-main) are presented for each parameter and each condition (n = 15).

The impact of the three parameters on QS_mobile.GL_ was very variable according to the S amounts and the timing conditions (Fig 5A and 5B). Both the PRCC and Sobol indices indicated the strong influence of the variation in RUE on QS_mobile.GL_. The PRCC values highlighted similar patterns of sensitivity of QS_mobile.GL_ to variations in RUE, β and SLA depending on the S amounts and timing conditions (Fig 5A). PRCC values were negative and tended to get closer to 0 with low S amounts under the GS30 and the GS30+60 conditions or with high amounts of S under GS60 conditions. Therefore, the higher the S amounts when supplied at GS30 or GS30+60, the higher the negative impact of the variation in RUE, β and SLA on QS_mobile.GL_. In contrast, under GS60 conditions the impacts of the variations in RUE, β and SLA was not so dependent on the S amounts because the PRCC values were more stable, with mean values of *ca*. -0.79, -0.54 and -0.31 for RUE, β and SLA, respectively, with all conditions combined. Regarding the Sobol indices, RUE was the most influential parameter in terms of total and main effects (Fig 5B) for most of the S-amounts and timing conditions, except for the following fertilisation designs: 300U at GS60 (in this case β was the most influential), and 37.5U and 50U at GS30+60 (in these cases RUE and β were equally influential). The total index for β decreased concomitantly with the increase in the S amount at GS30 and GS30+60 (Fig 5A and 5B) as also observed for SLA (Fig 5B), which contradicts the PRCC indices. However, the impact of the variation in the three parameters was less influenced by the S amount when provided at GS60 with its lower Sobol indices, except in the case of 300U. The interaction indices varied for the three parameters across all S amounts and timing conditions. The interaction values decreased as the S amounts increased under GS30 and GS30+60 conditions when the S amounts were higher than 20U, but the same trend was not seen at GS60 where lower and stable interaction indices across the S amounts were observed.

### Identification of optimised parameter combinations under different S-fertilisation strategies

The PRCC analyses were conducted from a random draw of 200 combinations of the 3 parameters associated with an output value. Therefore, we could identify the most relevant plant parameter combinations (i.e. optimised parameter combinations) that enabled the best plant performances under the different fertilisation strategies tested. The best plant performances were associated with high biomass (TDW) and green leaf area (LAI_GL_) to maximise plant development and light interception and high levels of mobile S (QS_mobile.GL_) to allow adequate S storage in pods. The condition where 75U of S was applied at GS30 was used as the S supply reference condition (RC). The best plant performances obtained under RC were associated to optimised parameter combinations (RC_opt_) which were thus compared to other optimised parameter combinations under the other S supply conditions (Table 3).

**Table 3:** Highest values of the three outputs (TDW, LAI_GL_ and QS_mobile.GL_) for each of 15 S-supply conditions obtained with optimized parameters combinations.

	TDW				LAI_GL_				QS_mobile.GL_			
Total S rate supply (kg.ha^-1^)	Δ TDW	RUE	SLA	β	Δ LAI_GL_	RUE	SLA	β	Δ QS_mobile.GL_	RUE	SLA	β
**RC**_**opt**_ *(75 U of SO*_*3*_ *at GS30)*	**56.4**	**4.58**	**0.016**	**0.54**	**6.49**	**2.59**	**0.018**	**0.65**	**25.68**	**1.61**	**0.022**	**0.11**
**Supply at GS30 (bolting)**												
20	- 4%	0%	+ 83%	- 19%	- 24%	- 35%	+ 79%	- 26%	- 89%	=	- 61%	+ 17%
37.5	- 2%	- 3%	+ 91%	- 15%	- 6%	- 33%	+ 79%	- 3%	- 71%	+ 13%	- 53%	+ 49%
50	=	- 1%	+ 53%	+ 26%	- 2%	- 20%	+ 8%	- 1%	- 48%	=	- 45%	+ 83%
300	+ 13%	- 1%	+ 83%	+ 5%	+ 8%	+ 55%	- 43%	+ 3%	+ 413%	+ 2%	- 49%	+ 60%
**Supply at GS60 (flowering)**												
20	- 28%	- 4%	+ 110%	+ 25%	- 53%	- 31%	+ 87%	- 14%	- 75%	+ 8%	- 19%	+ 127%
37.5	- 27%	0%	+ 65%	- 33%	- 50%	- 21%	+ 27%	+ 8%	- 42%	+ 5%	- 51%	+ 107%
50	- 28%	- 2%	+ 2%	- 10%	- 46%	- 31%	+ 79%	+ 4%	- 19%	+ 7%	+ 19%	+ 95%
75	- 24%	0%	+ 97%	+ 11%	- 44%	- 33%	+ 71%	- 16%	+ 31%	+ 3%	- 58%	+ 181%
300	- 20%	- 1%	+ 97%	+ 3%	- 35%	- 27%	+ 81%	- 21%	+ 436%	+ 4%	- 61%	+ 130%
**Fractioned S supply GS30+60**												
20	+ 6%	- 2%	+ 97%	+ 4%	- 37%	- 1%	+ 90%	+ 4%	- 98%	+ 97%	- 63%	+ 178%
37.5	+ 10%	0%	+ 41%	+ 20%	- 7%	- 35%	+ 42%	+ 5%	- 77%	+ 5%	- 63%	+ 46%
50	+ 12%	- 2%	+ 69%	- 44%	+ 2%	- 33%	- 2%	- 5%	- 41%	+ 6%	- 60%	+ 9%
75	+ 12%	- 1%	- 17%	+ 7%	+ 9%	- 13%	+ 2%	+ 1%	+ 7%	+ 2%	- 43%	+ 17%
300	+ 18%	0%	+ 10%	+24%	+ 13%	+ 51%	- 53%	+ 4%	+ 468%	+ 6%	- 60%	+ 32%

Figures in bold give the values of the outputs and parameters under the reference condition (RC) with optimized combinations (RC_opt_) which corresponds to 75 U of SO_3_ at GS30. The other figures are given as the proportion of increase or decrease compared to the values under RC_opt_. The equal symbol (=) means there was no difference in values with RC_opt_.

#### Early and fractioned S supplies allowed the highest biomass production but different optimised plant parameter combinations

When applied once at GS30, lower amounts of S than the reference condition (<75 U) led to small TDW variations even when the parameter combinations were optimised (-4% to -2% of RC_opt_). A higher S supply rate than in the RC_opt_ (>75 U) resulted in a 13% increase in TDW with optimisation of the parameter combinations (Table 3). Whatever the S supply rate (sub and supra), the RUE values remained almost unchanged to those under RC_opt_ with a maximum threshold value of 4.58 MJ.m^-^^2^ under RC_opt_. In contrast, the SLA and β values were drivers of TDW variation because their variations were significant, ranging from +53% to +91% for SLA, irrespective of the amount of S and from -19% to +26% for β with increases only observed when S amount were above the RC_opt_. Under 50 U, the TDW remained unchanged if the SLA increased by 53% to RC_opt_ and β increased by 25% compared to RC_opt_ (75 U at GS30). When applied at GS60, there was a decrease in TDW from -20% to -28% compared to RC_opt_ under all levels of S supply. When S was fractioned (GS30+60), the TDW increased from 6% to 18% under the extreme S supplies (20 and 300 U respectively) compared to RC_opt_ (75U at GS30). The fractioned condition led to the highest TDW increase even under sub amounts (50U) if SLA increased by 69% and β values were almost two times lower than under RC_opt_ (Table 3).

#### The fractioned S supply allowed substantial increases in LAI_GL_ with moderate changes in the plant parameters

When S was supplied at GS30, LAI_GL_ decreased by 24% to 2% compared to RC_opt_ for S amounts below RC (<75 U) and increased by 8% with 300 U. The SLA values were higher whereas the RUE and β values were lower than under the RC_opt_, except under 300 U where SLA decreased by 43%, unlike RUE and β, which increased by 55% and 3%, respectively. Whatever the S amount, β was the least impacting driver with the smallest variations (-26% to 3%) while the ranges of values for RUE and SLA were much wider, with -35% to 55% for RUE and -43% to 79% for SLA (Table 3). For S supplies at GS60, despite optimised parameter combinations, LAI_GL_ decreased regardless of the S amount, but as shown in Fig 2B the higher the S amount, the lower the decrease. The RUE values also decreased at GS60 and the SLA values increased compared to their values under RC_opt_ (from -33% to -21% for RUE and from 27% to 87% for SLA, Table 3).

Under the fractioned supply (GS30+60), LAI_GL_ increased with 50U, 75U and 300U by 2%, 9% and 13%, respectively, with optimised parameters combinations. For S amounts lower than RC (<75U), the optimised parameter combinations led to a reduction in LAI_GL_ (or an insignificant increase, as observed under 50U).

#### High QS_mobile.GL_ was driven by the S amount, whatever the timing of supply, with little impact on optimised parameter combinations

Whatever the timing of supply (at GS30, GS60 or GS30+60), QS_mobile.GL_ decreased for S amounts lower than the RC (<75 U) and increased when the S supply was higher than RC. As expected, the increase was significant for the 300 U supplies at GS30+60 (up to 468% higher than under RC_opt_). The RUE and β values increased regardless of the S supply and timing compared to RC, while the SLA values decreased, except at GS60 with 50U. However, RUE values displayed the lowest increases compared to SLA and β for a given S condition. Interestingly, for amounts lower than RC (< 75U), the increase in QS_mobile.GL_ was higher when S was supplied once at GS60 (+31%) than twice under GS30+60 (+7%).

## Discussion

### Plant parameter rankings according to their impacts on plant performance under different S-fertilisation strategies

The three parameters (RUE, SLA and β) selected for the SAs are associated with C-metabolism and functioning. They were shown to be impacted by S supply [2], thus leading to conclude to interactions between the C and S-related processes within the model. Our analysis highlighted that impacts on the variations (range and direction) of the outputs (TDW, LAI_GL_ and QS_mobile.GL_) were parameter-specific. These impacts have been summarised in Table 4, which combines conclusions from both the PRCC and Sobol indices.

**Table 4:** Ranking of the 3 parameters according to their respective impact on TDW, LAI_GL_ and QS_mobile.GL_ according to S-supply condition.

Output variable	Parameter ranking according to its impact	S-fertilization strategy	Correlation	Dynamics
**TDW**	RUE > β ≈ SLA	whatever S amount and timing of application	RUE, SLA and β positively correlated	• Stable impact of increased RUE whatever S amount and timing • More stable impact of increased SLA and β for supplies at GS60
**LAI**_**GL**_	SLA > β > RUE	with 20U at GS30 and at GS30+60	RUE, SLA and β positively correlated	• Stable impact of increased β whatever S amount and timing • Lower impact of increased SLA with higher S rate at GS30 and GS30+60 • Slightly higher impact of increased RUE with S rate at GS30 and lower impact of increased RUE with S rate up to 75U at GS30+60
	β > SLA > RUE	other conditions		
**QS**_**mobile.GL**_	β > RUE > SLA	with 37.5U at GS30 with 300U at GS60	RUE, SLA and β negatively correlated *except RUE and β with 20U at GS30 and with 50U at GS30+60*	• Higher impact of increased RUE, SLA and β with S rates especially at GS30 and GS30+60
	RUE > SLA > β	with 20U and 300U at GS30		
	RUE > β > SLA	other conditions		

This synthesis including results of the Sobol (main indices) and PRCC analyses.

The RUE was an important driver of the variation in TDW and QS_mobile.GL_. It was the most impacting parameter for TDW with a positive trend that was stable across all S supply conditions (amounts and timing), meaning that increasing its value leads to higher biomass at the onset of pod formation. There is a direct correlation between RUE and TDW from Monteith’s model [38], which is used in the SuMoToRI model [2]. In contrast, the two other parameters, β and SLA, had very little impact on TDW, which made sense because they directly relate to the leaves. According to the total Sobol indices, the allocation of carbohydrates to the leaves (β) was slightly more influential than the SLA (with values ranges of 0.072–0.12 for β and 0.041 to 0.094 for SLA). Because no interactions were observed, the effective impact of a given parameter’s variation was not influenced by the other parameters, which confirms that they each described distinct processes (i.e. biomass production vs. biomass allocation).

The SAs also revealed that the most impacting parameters on LAI_GL_ were β, and to a lesser extent, SLA and RUE. As expected, increased C-allocation to the leaves (β) and SLA favoured greater leaf area expansion while higher RUE had little impact. While the effect of β was stable with increasing S-amounts when S was provided at bolting alone or at bolting and flowering, the effects of SLA and RUE were influenced by the S amounts in these timing conditions. This was consistent with prior observations during the model calibration and evaluation steps that (i) led to different RUE values according to the S-supply conditions [2] and (ii) pointed out that the SLA decreased under S-limiting conditions, meaning that the leaf thickness was greater (Brunel-Muguet, unpublished). This leaf plasticity was showed to increase the photosynthetic activity, thus setting a compensatory mechanism against drastic S limitation, which is known to impair photosynthesis and C metabolism [39].

Regarding the amount of mobile S within the leaves (QS_mobile.GL_), the respective impact of the parameters varied according to S supply conditions. The Sobol indices indicated that RUE was the main driver followed by β and SLA. Increasing RUE, β and SLA led to lower QS_mobile.GL_, which was amplified by increases in S fertilisation when supplied either once at bolting or twice under the fractioned condition. In contrast, the impact of the three parameters was much lower under all levels of S supply at flowering. As observed, increasing the RUE favoured biomass production and consequently leaf expansion. This in turn increased the leaf S-structural requirements and thus depleted the mobile S amounts stored within the leaves. Another reason that could account for these effects is the dilution of S within the plant. As the size of the source organs increases, the concentration of S is diluted, leading to depletion of S mobile reserves. This effect of increased RUE was observed for optimal timing i.e. at bolting and in the fractioned condition. It was also lower as the S supplies decreased.

### A preliminary approach to design ideotypes adapted to specific S-fertilising strategies

In our study, we made the assumption that ideal parameter combinations combined with adequate S-fertilisation strategies should offer the most efficient plant status for achieving the reproductive phase and then high yield. Our analyses revealed that the optimised combinations for the best performance at the onset of pod formation (i.e. high biomass and green leaf area to maximise plant development and light interception and high mobile S to allow adequate S storage for growing pods) were specific to each output, thus meaning that there will be trade-offs to attain them in combination (Table 3). For instance, the optimal RUE value for the highest TDW (for the RC_opt_) is 4.58, whereas it is 2.59 to attain the highest LAI_GL_. Moreover, in some cases, it is crucial to consider several parameters together because of their strong interactions, as illustrated for QS_mobile.GL_ (Fig 5B).

Table 4 synthesises the parameter rankings for a given output (i.e. plant biomass, leaf expansion, S storage) according to the S supply strategies. It indicated that the most beneficial parameter for targeting would differ depending on whether we intended to improve plant biomass, leaf expansion or S storage within the leaves with respect to the S-fertilisation designs. Indeed, RUE was the driver of plant biomass and its impact was stable regardless of amount and timing of S-fertilisation (stage and fractioning). Leaf expansion was mainly boosted by increased C allocation to the leaves (β) and to a lesser extent by increased specific leaf area and RUE, in all S-fertilisation designs apart from low S amounts (20U) when supplied at bolting (GS30) or in the fractioned condition (GS360+690). Impacts of increased RUE and SLA differed depending on the S-fertilisation designs. While the impact of SLA was higher with increasing rates of S-fertilisers at flowering (GS60), the impact of RUE was more pronounced in the fractioned condition with extreme amounts (20 and 300U).

These SAs revealed that the association between high biomass or leaf area, and S storage for growing was not trivial because the plant parameter drivers were distinct and the intensity of their impact was also dependent on the S fertilisation designs. Genotypes with high radiation efficiency, large light interception surfaces (per biomass unit) or high leaf biomass allocation would benefit from early and fractioned S-fertilisation strategies.

### Towards new S management strategies

These results confirm the need to think about new designs or cultural practices for S fertilisation. First, they highlighted the negative impact of delayed fertilisation (i.e. at flowering). When applied at this stage (GS60), a higher S supply could not compensate for the earlier reductions in vegetative growth. In addition, our results indicated the relevance of fractioning S supply compared to the conventional single supply at bolting, and thus broadened fertilisation schemes. In our conditions, S-fractioning led to similar plant performances at the onset of pod formation. This strategy also prevented excessive sulfate being stored in the leaves and then its potential loss when leaves detached. From an ecological and economic perspective, fractioning would allow better nutrient adjustment rather than the conventional single supply and thus help in preventing over fertilisation. Nevertheless, our results showed that even under the most optimised plant parameter combination, the increase in TDW of around 13% under the fractioned condition was obtained with the highest S supply, which ultimately moderates the sustainable effect of fractioning. In addition, it is known that S nutrition is closely related to N nutrition in many species like oilseed rape [21,22,40], wheat [41,42] and maize [43]. Fismes *et al*. [44] reported that in oilseed rape, the balance between the S and N rate determines their use efficiencies, which are synergistic at optimum rates and antagonistic at excessive levels of a single element. S fertilisation is known to improve N use efficiency and to maintain high seeds quality [45], thus highlighting the importance of balanced N:S ratio. In our study, the fifteen S fertilisation conditions were provided with non-limiting N supply. For the lowest S supply conditions, the N:S ratio was not as well balanced as for the conditions with the conventional S supply, which could impact crop performances. N inputs are drivers of this ratio meaning they can modulate crop responses to S supply. In this context, implementing S: N ratio thresholds in the model could help monitor S fertilisation to better monitor effective S assimilation, which is partly determined by N availability.

## Conclusion

A better understanding of the relationship between specific model parameters and crops at the onset of pod formation is essential to adjust cultural practices under contrasting S fertilisation management. For this purpose, we used GSAs to rank driver model parameters including RUE, β and SLA according to their impact on main output variables related to growth, leaf expansion and S remobilisation. In our study, we compared these plant parameters combinations to distinguish S-fertilisation designs. This enabled the best S-fertilisation designs to be determined under specific plant parameters combinations. This approach underscores the advantages of using adapted varieties for a specific fertilisation context. Our study also highlighted the importance of S fertilisation management in terms of the amount supplied or the timing of the application (stage and fractioning). We showed that supplying more than the recommended inputs did not lead to proportional improvements in plant biomass or leaf expansion and that delaying supply until flowering had a strong negative impact. If fractioning designs seem to be competitive with the conventional single supply (75U SO_3_ at the end of winter), significant increased performance would require higher S rates. These findings reinforce the need to develop tools to measure plant S status and soil S availability *in situ*, which would help in adjusting fractioned S supplies throughout the crop cycle.

## Supporting information

S1 Fig Climatic data used for model simulation and sensitivity analyses. Daily mean temperature in °C (A) and daily mean Photosynthetically Active Radiation (PAR) in MJ.m^-2^ (B) in Saint-Martin-de-Hinx averaged over 2005 to 2015 (source: CLIMATIK https://intranet.inra.fr/climatik_v2) for the simulated periods.
(TIF)
