# Supplementary figures and images for: Sensitivity analyses for improving sulfur management strategies in winter oilseed rape
Source: PLoS One. 2018 Sep 20;13(9):e0204376. doi: 10.1371/journal.pone.0204376 (PMC6147610; doi:10.1371/journal.pone.0204376)

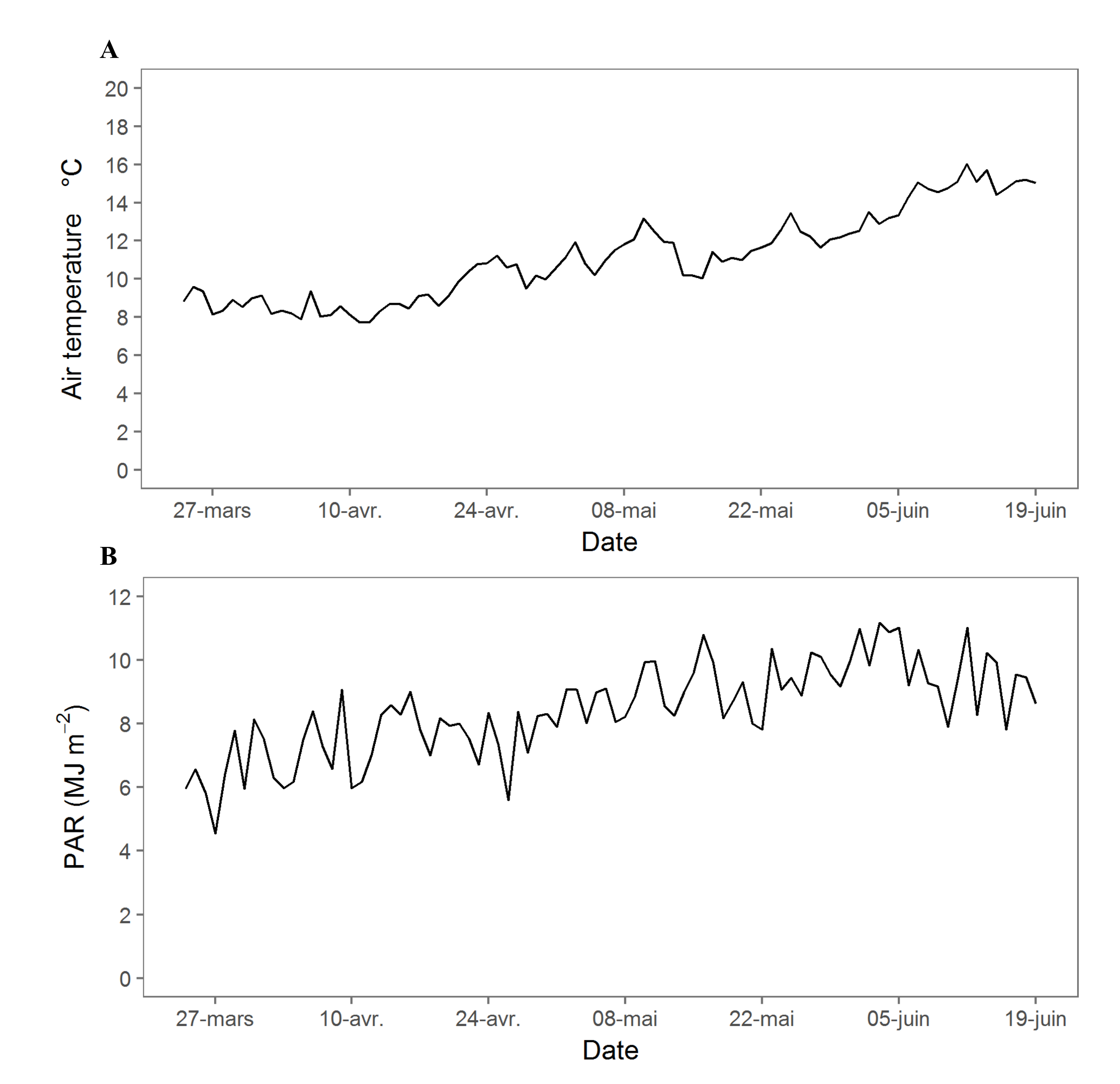

Supplement: S1 Fig — Daily mean temperature in °C (A) and daily mean Photosynthetically Active Radiation (PAR) in MJ.m-2 (B) in Saint-Martin-de-Hinx averaged over 2005 to 2015 (source: CLIMATIK https://intranet.inra.fr/climatik_v2) for the simulated periods. (TIF) [file pone.0204376.s001.tif]
